# Short-Term Aggregated Residential Load Forecasting of Low-Voltage Distribution Networks Based on Graph Neural Networks and K-Means Clustering

**DOI:** 10.3390/s26154978

**Published:** 2026-08-06

**Authors:** Fujia Han, Ji Qiao, Hao Yu, Zibo Wang

**Affiliations:** Department of Artificial Intelligence Research, China Electric Power Research Institute Co., Ltd., Beijing 100192, China; hanfujia@epri.sgcc.com.cn (F.H.); yuhao9663@163.com (H.Y.); wangzibo@epri.sgcc.com.cn (Z.W.)

**Keywords:** advanced metering infrastructures, residential load forecasting, temporal correlation, spatial correlation, graph neural networks, K-means clustering, spatial–temporal graph

## Abstract

With the rapid development and widespread deployment of advanced metering infrastructures, massive amounts of fine-grained data about aggregated residential load have been collected by power network operators, further helping improve forecasting accuracy. Accurate aggregated residential load forecasting plays an increasingly important role in various interactions between power networks and electricity customers as well as in maintaining the continuous balance between electricity supply and demand. However, most existing short-term aggregated residential load forecasting methods only take into consideration the temporal correlation of historical load profiles, ignoring the potential spatial correlation between electricity consumption behaviors of adjacent residential customers. Thus, to fill this gap, this paper proposes a short-term aggregated residential load forecasting method based on graph neural networks and K-means clustering. Specifically, K-means clustering is firstly used to divide residential customers into different groups according to the similarity of their electricity consumption behaviors. Then, the spatial–temporal graph series for aggregated residential load forecasting is constructed, based on the number of groups of residential customers, the aggregated historical load profile of each group of residential customers, and the correlation between the aggregated historical load profiles of different groups of residential customers. Finally, adaptive spatial–temporal synchronous graph convolutional networks are applied to perform short-term aggregated residential load forecasting. The proposed method is evaluated on a real-life Irish residential load dataset, and the experimental results demonstrate that it can improve forecasting accuracy significantly in comparison with a number of traditional benchmark methods.

## 1. Introduction

As the modern power network is gradually transitioning towards the smart grid with growing penetration of renewable energy generation, especially distributed generation, aggregated residential load forecasting is playing an increasingly important role in interactions between power network operators and residential customers, efficient and cost-effective network operations, and household energy consumption optimizations [1]. In addition, as load aggregators and virtual power plants have constantly been introduced into power distribution networks, a variety of electricity customers are capable of participating in electricity market transactions and interactions more proactively. Thus, as an important part of the transaction and interaction process, aggregated residential load forecasting is the basic prerequisite for pricing decisions in the electricity market. However, due to the complicated influences of weather conditions and electricity prices, as well as personalized electricity consumption habits of residential customers, electricity consumption patterns of residential customers have high volatility and uncertainty, therefore posing a huge challenge to aggregated residential load forecasting [2,3]. With the widespread deployment of advanced metering infrastructures across the modern power network, for example, smart meters, a huge amount of monitoring data regarding the electricity consumption behaviors of residential customers has been generated, thereby significantly facilitating aggregated residential load forecasting.

At present, a number of researchers have primarily focused on system-level, substation-level, and feeder-level load forecasting methods based on artificial intelligence technologies, such as machine learning and deep learning [4,5,6,7,8,9,10,11,12,13]. Specifically, machine learning algorithms include support vector regression, random forest, and artificial neural networks, while deep learning algorithms include recurrent neural networks, convolutional neural networks, and deep feed-forward neural networks [14,15]. In general, due to the relatively weak volatility and the comparatively obvious periodicity and seasonality of system-level, substation-level, and feeder-level loads, a majority of existing system-level, substation-level, and feeder-level load forecasting methods based on artificial intelligence technologies are capable of obtaining high forecasting accuracy. In particular, during recent years, an increasing number of researchers have applied deep learning algorithms to system-level, substation-level, and feeder-level load forecasting in order to improve forecasting accuracy [16,17]. The main reason for this is that compared to machine learning algorithms, deep learning algorithms have the superior ability of mining complicated non-linear relationships in a large amount of data, therefore significantly enhancing the expression effect regarding sample distribution patterns.

By contrast, only a few efforts have been made towards aggregated residential load forecasting in recent years [18,19,20,21,22,23,24,25,26]. Currently, aggregated residential load forecasting methods are generally categorized into bottom-up forecasting methods and top-down forecasting methods. However, most existing aggregated residential load forecasting methods have focused on mining the temporal correlation of residential load, but have ignored the potential correlation among electricity consumption behaviors of residential customers, thus limiting further improvement of the forecasting accuracy. For instance, residential customers in the same area tend to share the same influence factors, including geographical space, weather conditions, holiday information, and electricity price policies. As a result, there is commonly a certain degree of potential correlation among electricity consumption behaviors of residential customers. In recent years, graph neural networks, as an emerging type of neural network, can effectively learn graphs and have the superior ability to simultaneously mine the feature information within nodes and the correlation information among nodes. So far, graph neural networks have been widely applied to the research fields of solar power forecasting, wind power forecasting, and traffic forecasting and are thereby regarded as a new solution to aggregated residential load forecasting [27,28,29,30].

Thus, in order to solve the above issue, this paper presents a novel short-term aggregated residential load forecasting method, which is capable of taking into consideration both the temporal correlation of residential load and the spatial correlation between electricity consumption behaviors of adjacent residential customers. The main contributions of this paper are summarized as follows:

- A short-term aggregated residential load forecasting method is proposed to further improve the forecasting accuracy, based on an effective combination of graph neural networks and K-means clustering. To be specific, graph neural networks are employed in order to make full use of the spatial–temporal correlation in aggregated residential load forecasting.
- Two different spatial–temporal graph series constructing methods for short-term aggregated residential load forecasting are developed based on the Pearson correlation coefficient. More specifically, in the spatial–temporal graph series, the node is utilized to express the temporal correlation of residential load, while the edge is utilized to express the spatial correlation between electricity consumption behaviors of adjacent residential customers.
- The proposed short-term aggregated residential load forecasting method is evaluated and compared with several benchmark methods on a real-life Irish residential load dataset, and the experimental results show that it can obtain the higher forecasting accuracy, in terms of root mean square error (RMSE) and mean absolute error (MAE), as well as mean absolute percentage error (MAPE).

The remainder of this paper is organized as follows. Section 2 reports a comprehensive literature review on load forecasting. Section 3 elaborates adaptive spatial–temporal synchronous graph convolutional networks (ASTSGCNs) as well as K-means clustering and further details the integration of graph neural networks with K-means clustering for short-term aggregated residential load forecasting. In Section 4, the presented aggregated residential load forecasting method is evaluated on a real-life Irish residential load dataset. Finally, the conclusions are drawn and the future work is pointed out in Section 5.

## 2. Related Work

Currently, numerous research works have already been presented in the field of system-level, substation-level, and feeder-level load forecasting [4,5,6,7,8,9,10,11,12,13]. For instance, Liu et al. [4] proposed a distributed system-level load forecasting method based on local weather information. It firstly partitions the bulk power system into some subnets based on local weather information, and then establishes separate forecasting models for subnets from a load forecasting model base, finally developing a system-level load forecasting model to aggregate local load forecasts. Another short-term system-level load forecasting method based on modified deep residual networks was presented in [6]. This method integrates domain knowledge to improve its forecasting performance and utilizes a two-stage ensemble strategy to enhance its generalization capability. Similarly, Li et al. [8] designed a novel wavelet-based ensemble method for short-term system-level load forecasting with hybrid neural networks and feature selection for increased forecasting performance. In addition, Rafiei et al. [10] developed a hybrid method for probabilistic system-level load forecasting, including a generalized extreme learning machine for training an improved wavelet neural network, wavelet preprocessing and bootstrapping. This method takes data noise uncertainties into account while outputting the load probabilistic interval. In [11], an efficient hierarchical method for short-term load forecasting at the distribution level was proposed. Specifically, the load of the root node at any user-defined subtree is firstly forecasted by a wavelet neural network with appropriate inputs, and child nodes categorized as “regular” and “irregular” based on load pattern similarities are then forecasted separately. This method is capable of capturing load characteristics of nodes at different levels, and it takes advantage of pattern similarities between a parent node and its child nodes. Cao et al. [12] presented a hybrid ensemble deep learning method for substation-level load forecasting, which effectively combines multiple separate ensemble strategies and the K nearest neighbor classification algorithm to adaptively determine the weight of each model. Moreover, Dong et al. [13] developed a long-term distribution feeder load forecasting method based on sequence prediction, which is able to seamlessly integrate top-down, bottom-up, and sequential information hidden in multi-year data.

To sum up, as system-level, substation-level, or feeder-level load tends to be comparatively smooth and stable, it is quite easy to obtain high forecasting accuracy on system-level, substation-level, or feeder-level load forecasting, regardless of applying traditional machine learning or deep learning methods.

By contrast, there have been only a small number of research works in the area of aggregated residential load forecasting during recent years [18,19,20,21,22,23,24,25,26]. At present, artificial intelligence-based aggregated residential load forecasting methods can be generally classified as bottom-up and top-down. To be specific, bottom-up forecasting methods firstly aggregate the load of each individual residential customer and then they model and predict the aggregated load of all residential customers. Ge et al. [18] proposed a short-term aggregated residential load forecasting method combining the maximum information coefficient, factor analysis, gray wolf optimization, and generalized regression neural networks. Within this method, to screen and decrease the dimension of the multiple-input features, the maximum information coefficient is firstly used to quantify the non-linear correlation between the load and input features, and to eliminate the ineffective features, and then factor analysis is used to reduce the dimension of the screened input features on the premise of preserving the main information of input features. Afrasiabi et al. [19] presented a direct method for conditional probability density forecasting of aggregated residential load based on a deep mixture network, which can provide comprehensive information about future uncertainties of aggregated residential load. Likewise, a novel online ensemble learning method for aggregated residential load forecasting was developed by combining batch and online learning in [20]. Compared to bottom-up forecasting methods, top-down forecasting methods firstly divide all residential customers into different groups and then predict the load of each group of residential customers respectively, finally integrating the load forecasting result of each group of residential customers to obtain the predicted aggregated load of all residential customers. For example, Park et al. [22] designed a day-ahead aggregated residential load forecasting scheme based on characteristic load decomposition. This scheme firstly applies a hybrid strategy to obtain separate forecasts for decomposed characteristic load profiles, and then sums them up to predict the aggregated residential load. In [24], a bottom-up aggregated residential load forecasting method was proposed on the basis of hierarchical clustering. To be specific, this method uses random forest to obtain the forecast of each subset of residential customers and then sums up with a convex expert aggregation strategy to predict the aggregated residential load. Also, Wang et al. [25] presented an ensemble forecasting method for aggregated residential load, which is capable of producing multiple forecasts by different groupings of subprofiles and provides the final forecast based on a set of optimal weights.

Although a variety of forecasting methods for aggregated residential load have been developed, they are unable to take full advantage of both the temporal correlation of residential load and the potential correlation among electricity consumption behaviors of residential customers, thus limiting further improvement of the forecasting accuracy. Our proposed method is capable of bridging the gap by integrating graph neural networks with K-means clustering in order to mine the spatial–temporal correlation in aggregated residential load forecasting effectively.

## 3. Methodology

### 3.1. Adaptive Spatial–Temporal Synchronous Graph Convolutional Network

#### 3.1.1. Localized Spatial–Temporal Graph

A spatial graph represents the relationship between different nodes in the spatial dimension, generally defined as:

(1) G=(V, E, A)

where the spatial graph *G* consists of a set of nodes *V* and a set of edges *E*, $N=\left|V\right|$ is the number of nodes in the spatial graph, and $A\in R^{N\times N}$ is the adjacency matrix of the spatial graph, which quantifies the relationship between nodes. As a novel type of graph neural network, ASTSGCNs are capable to simultaneously mine the temporal and spatial correlations of localized spatial–temporal graphs.

**Figure 1:**
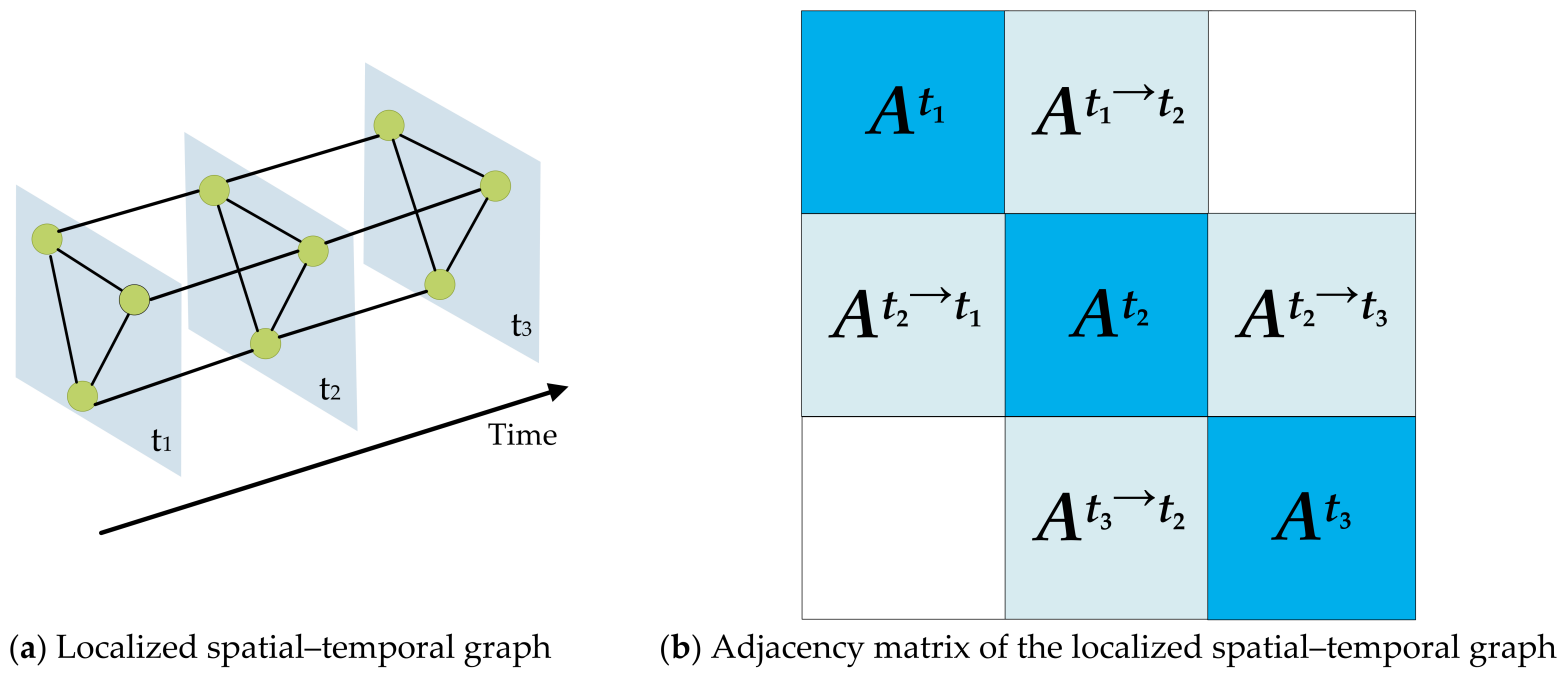
Schematic diagram of the localized spatial–temporal graph and its adjacency matrix.

Figure 1 shows the localized spatial–temporal graph and its adjacency matrix. A localized spatial–temporal graph is generally constructed based on three continuous spatial graphs, which is able to connect each node with itself at the previous and next time steps. The intrinsic correlation of the localized spatial–temporal graph is represented by the adjacency matrix $A^{\prime }\in R^{3N\times 3N}$. If two nodes connect with each other in the localized spatial–temporal graph, the corresponding value in the adjacency matrix is set as one. The adjacency matrix of the localized spatial–temporal graph is formulated as follows [31]:

(2) $$A^{\prime }[i,\;j]=\left\{\begin{array}{c} 1,\;\;\mathrm{if}\;v_{i}\;\mathrm{connects}\;\mathrm{to}\;v_{j}\\ 0,\;\;\;\;\mathrm{otherwise}\; \end{array}\right.$$

where $A^{\prime }[i,\;j]$ is the *j*^th^ element of the *i*^th^ row in the adjacency matrix of the localized spatial–temporal graph, and *v_i_* is the node *i* in the localized spatial–temporal graph. The adjacency matrix $A^{\prime }$ contains 3*N* nodes. $A^{t_{i}}$ is the adjacency matrix of the spatial graph at the time step *t_i_*, and $A^{t_{i}\to t_{j}}$ denotes the connection between each node and itself at the time step *t_i_* and the time step *t_j_*.

#### 3.1.2. Adaptive Adjacency Matrix

For adaptive spatial–temporal synchronous graph convolutional networks, the adjacency matrix $A^{\prime }$ of the localized spatial–temporal graph determines the weights of the aggregation of the graph convolutional operation. Thus, in order to enhance the aggregating ability of the graph convolutional operation, a learnable mask matrix $W_{mask}\in R^{3N\times 3N}$ is added to improve the adjacency matrix $A^{\prime }$ of the localized spatial–temporal graph. To be specific, the adaptive adjacency matrix ${A^{\prime }}_{mask}\in R^{3N\times 3N}$ of the localized spatial–temporal graph is obtained by the element-wise product between $W_{mask}$ and $A^{\prime }$, as shown below [31]:

(3) $${A^{\prime }}_{mask}=A^{\prime }⊗W_{mask}\in R^{3N\times 3N}$$

The elements of the learnable mask matrix $W_{mask}$ can be initialized in the following way [31]:

(4) $$W_{mask}[i,\;j]=\left\{\begin{array}{c} 1,\;A^{\prime }[i,\;j]\neq 0\\ 0,\;A^{\prime }[i,\;j]=0 \end{array}\right.$$

where $W_{mask}[i,\;j]$ is the *j*^th^ element of the *i*^th^ row in the learnable mask matrix.

#### 3.1.3. Spatial–Temporal Embedding

As the input of ASTSGCNs, the spatial–temporal graph series $X_{G}$ is shown as follows [31]:

(5) $$X_{G}=(X_{G}^{t-T+1},\;X_{G}^{t-T+2},\;\ldots ,\;X_{G}^{t})\in R^{N\times C\times T}$$

where $X_{G}^{t}\in R^{N\times C}$ is the graph signal matrix representing the observations of the spatial graph *G* at the time step *t*, *T* is the length of the spatial–temporal graph series, and *C* is the number of attribute features.

In order to enhance the ability of ASTSGCNs to mine the spatial–temporal correlation, a learnable temporal embedding matrix $T_{emb}\in R^{C\times T}$ and a learnable spatial embedding matrix $S_{emb}\in R^{N\times C}$ are created respectively through random initialization. After the training process is completed, these two embedding matrices will contain the necessary temporal and spatial information to help ASTSGCNs capture the spatial–temporal correlation effectively. They are added to the spatial–temporal graph series $X_{G}$ with the broadcast operation to obtain the new representation $X_{G+t_{emb}+s_{emb}}$ of the spatial–temporal graph series as follows [31]:

(6) $$X_{G+t_{emb}+s_{emb}}=X_{G}+T_{emb}+S_{emb}\in R^{N\times C\times T}$$

#### 3.1.4. Adaptive Spatial–Temporal Synchronous Graph Convolutional Layer

An adaptive spatial–temporal synchronous graph convolutional layer (ASTSGCL) normally consists of multiple adaptive spatial–temporal synchronous graph convolutional modules (ASTSGCMs), which are capable of capturing the localized spatial–temporal correlation. Specifically, an ASTSGCM is composed of a group of graph convolutional operations aggregating the features of each node with its neighbors at adjacent time steps. The input of the graph convolutional operation is the graph signal matrix of the localized spatial–temporal graph. Thus, the graph convolutional operation can be formulated as follows [31]:

(7) $$h^{\left(l\right)}=\sigma ({A^{\prime }}_{mask}h^{\left(l-1\right)}W+b)\in R^{3N\times C^{\left(l\right)}}$$

where $h^{\left(l-1\right)}\in R^{{3N\times C}^{\left(l-1\right)}}$ is the input of the *l*^th^ graph convolutional layer, $W\in R^{C^{\left(l-1\right)}\times C^{\left(l\right)}}$ and $b\in R^{C^{\left(l\right)}}$ are the learnable parameters of the *l*^th^ graph convolutional layer, $C^{\left(l-1\right)}$ and $C^{\left(l\right)}$ are the numbers of features of the input and the output of the *l*^th^ graph convolutional layer respectively, and σ is the activation function, which is normally represented by ReLU as follows:

(8) $$\mathrm{ReLU}(x)=\left\{\begin{array}{c} 0,\;x<0\\ x,\;x\geq 0 \end{array}\right.$$

**Figure 2:**
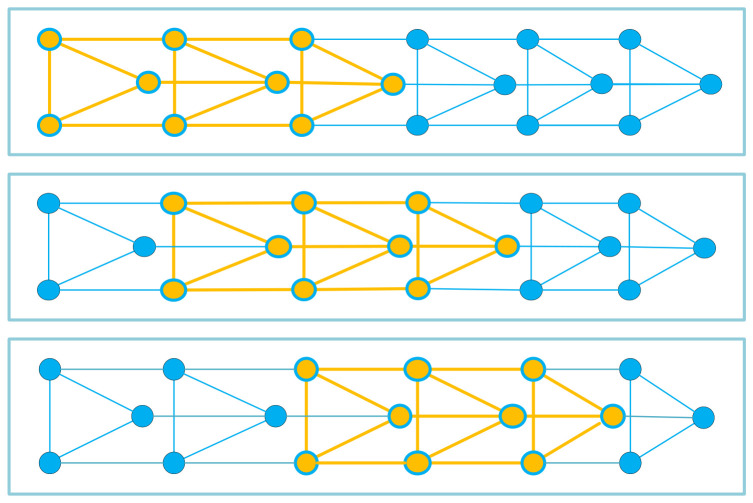
Schematic diagram of the sliding window.

In order to extract the long-range spatial–temporal features of the entire spatial–temporal graph series, a sliding window is used to cut out the spatial–temporal graph series into different periods sequentially. The schematic diagram of the sliding window is shown in Figure 2. Multiple ASTSGCMs are utilized to model different periods so that each ASTSGCM can focus on capturing the localized spatial–temporal correlation in the corresponding localized spatial–temporal graph. To be specific, spatial–temporal embeddings for the ASTSGCL are added at first. Then, the sliding window in the ASTSGCL is used to cut out the entire spatial–temporal graph series into *T*-2 localized spatial–temporal graphs. Finally, the ASTSGCL deploys *T*-2 ASTSGCMs on *T*-2 localized spatial–temporal graphs to capture the localized spatial–temporal correlation in these *T*-2 localized spatial–temporal graphs. In addition, the complex spatial–temporal correlation of the spatial–temporal graph series can be captured more effectively by stacking multiple ASTSGCLs, therefore enabling each node to contain the localized spatial–temporal correlation centered by itself.

#### 3.1.5. ASTSGCN Structure

**Figure 3:**
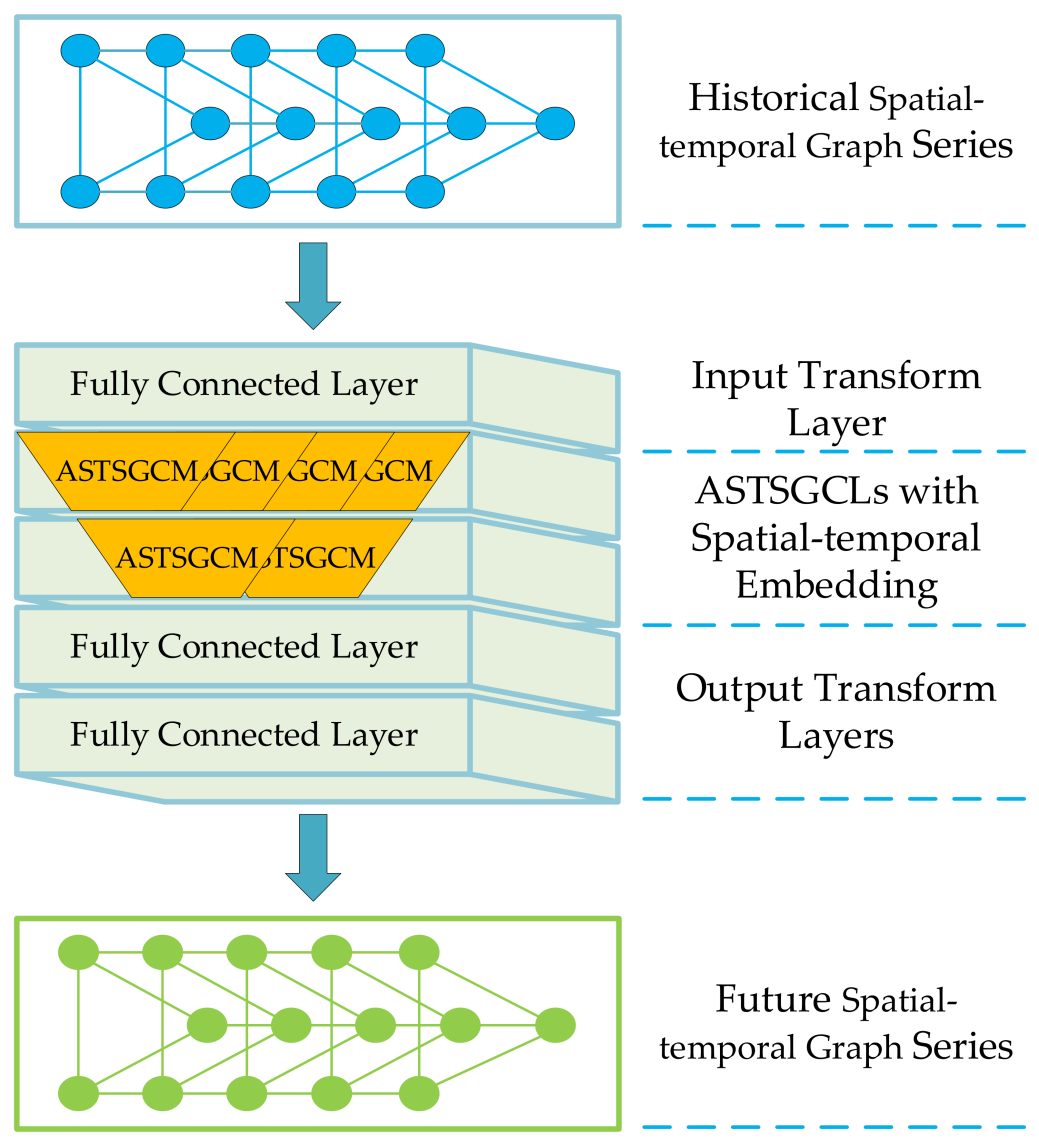
Structure of adaptive spatial–temporal synchronous graph convolutional networks.

The structure of adaptive spatial–temporal synchronous graph convolutional networks is shown in Figure 3. ASTSGCNs take historical spatial–temporal graph series as input and output future spatial–temporal graph series. Firstly, the historical spatial–temporal graph series is transformed from the low-dimensional feature space to the high-dimensional feature space through a fully connected layer. Then, stacked multiple ASTSGCLs with spatial–temporal embeddings capture the localized spatial–temporal correlation in the spatial–temporal graph series. Finally, the output of the stacked multiple ASTSGCLs is mapped to the future spatial–temporal graph series through two fully connected layers.

### 3.2. K-Means Clustering

Clustering is a common unsupervised learning technique in machine learning, which is intended for identifying the clusters of similar data samples in a dataset. Although numerous clustering methods have been developed, K-means clustering is still one of the most popular clustering methods in many fields due to its simplicity and robustness. As a result, K-means clustering is adopted to cluster residential load profiles in this paper.

Given a dataset $X=\left[x_{1},\;x_{2},\;x_{3},\;\cdots ,\;x_{n}\right]\in R^{p\times n}$, the goal of K-means clustering is to partition the data into *K* clusters so that $\mu_{k}\in R^{p}$ is the prototype associated with the $k^{\mathrm{th}}$ cluster for k=1, 2, 3, ⋯, K. A set of binary indicator variables $c_{ik}\in \left\{0,\;1\right\}$ is also introduced to represent the assignments, where $c_{i}={\left[c_{i1},\;c_{i2},\;c_{i3},\;\cdots ,\;c_{iK}\right]}^{T}$ is the $k^{\mathrm{th}}$ canonical basis vector in $R^{K}$ if and only if $x_{i}$ belongs to the $k^{\mathrm{th}}$ cluster.

Let $\mu ={\left\{\mu_{k}\right\}}_{k=1}^{K}$ be the cluster centers and $c={\left\{c_{i}\right\}}_{i=1}^{n}$ be the assignments of data samples. K-means clustering attempts to minimize the sum of the squared Euclidean distances of all the data samples to their assigned clusters [32]:

(9) $$f(c,\;\mu )={\sum_{i=1}^{n}\sum_{k=1}^{K}c_{ik}\left\|x_{i}-\mu_{k}\right\|}_{2}^{2}$$

where the objective function f(c, μ) is minimized by respectively updating the assignments c and the cluster centers μ in an iterative way. The update procedure is formulated as follows [32]:

Step 1: Minimize f(c, μ) over c while keeping μ fixed:

(10) $$\forall i=1,\;2,\;3,\;\cdots ,\;n:\;c_{ik}=\left\{\begin{array}{c} 1\;k=\arg\min_{j}{\left\|x_{i}-\mu_{j}\right\|}_{2}^{2}\\ 0\;\;\;\mathrm{otherwise}\; \end{array}\right.$$

Step 2: Minimize f(c, μ) over μ while keeping c fixed:

(11) $$\forall k=1,\;2,\;3,\;\cdots ,\;K:\;\mu_{k}=\frac{1}{n_{k}}\sum_{x_{i}\in S_{k}}x_{i}$$

where $S_{k}$ denotes the set of the data samples assigned to the $k^{\mathrm{th}}$ cluster, and $n_{k}$ denotes the number of the data samples $S_{k}$ contains.

In addition, in order to improve the performance of K-means clustering, the K-means++ algorithm is applied to initialize the cluster centers. Hence, the detailed steps of K-means clustering are presented in Algorithm 1.
**Algorithm 1:** K-means Clustering
**Input:** dataset X, number of clusters *K***Output:** cluster centers μ, assignments of data samples c
1         **Begin**2         Initialize the *K* cluster centers by the K-means++ algorithm3         **Repeat**4                 Assign each data sample to the closest cluster center using (10)5                 Update each cluster center using (11)6         **Until** no cluster members are reassigned to the new clusters or the decrease in the            objective function f(c, μ) is within the predefined limit7         **Terminate**

### 3.3. Short-Term Aggregated Residential Load Forecasting Based on Graph Neural Networks and K-Means Clustering

#### 3.3.1. Spatial–Temporal Graph Series for Short-Term Aggregated Residential Load Forecasting

Constructing the spatial–temporal graph series is the fundamental prerequisite for short-term aggregated residential load forecasting based on graph neural networks. It mainly consists of the following two steps.

Firstly, the node of the spatial graph of the spatial–temporal graph series for short-term aggregated residential load forecasting is constructed. As for a large number of residential customers, if each individual residential customer is regarded as a node of the spatial graph, it may cause a series of problems, such as high structural complexity of the spatial graph, excessive computation of the graph neural network, and serious forecasting instability due to significantly random electricity consumption behaviors of residential customers. Hence, based on the similarity of electricity consumption behaviors within residential customers, this paper utilizes K-means clustering to group the historical load profiles of all residential customers. Each group of residential customers is regarded as a node of the spatial graph, and the aggregated historical load profile of each group of residential customers is regarded as the features of the corresponding node at different time steps, therefore effectively enhancing the ability of the graph neural network to capture the electricity consumption pattern of each group of residential customers.

**Figure 4:**
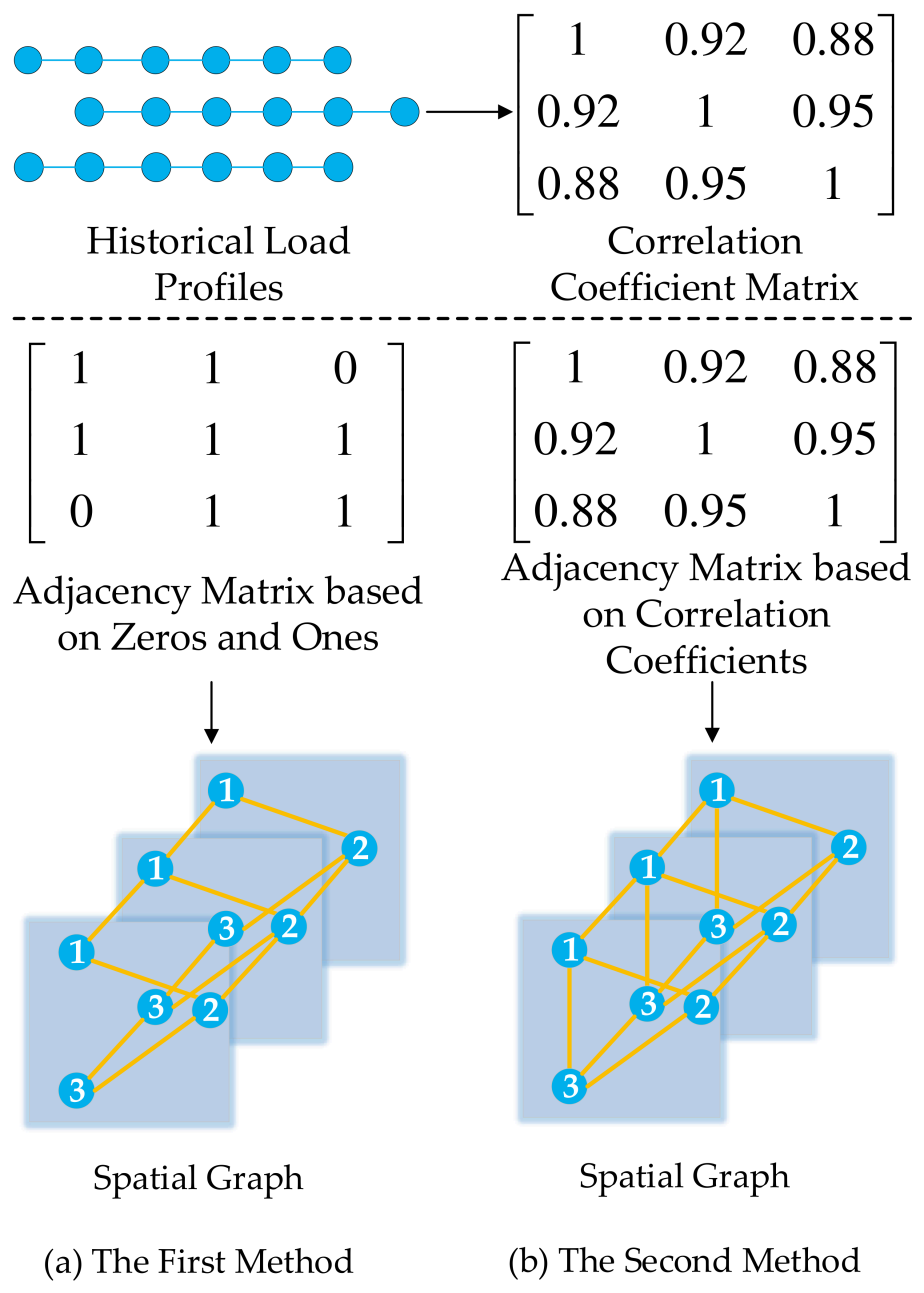
Schematic diagram of the adjacency matrices constructed by two different methods.

Secondly, the edge of the spatial graph of the spatial–temporal graph series for short-term aggregated residential load forecasting is constructed, which represents the element of the adjacency matrix of the spatial graph. This paper proposes two different methods for constructing the adjacency matrix of the spatial graph, as shown in Figure 4. The first method for constructing the adjacency matrix is shown in Figure 4a. To be specific, if the correlation coefficient between the features of the two nodes in the spatial graph is not less than a certain threshold, it is considered that there is a connection relationship between the two nodes, and the corresponding element of the adjacency matrix is set as one. Otherwise, it is considered that there is no connection relationship between the two nodes, and the corresponding element of the adjacency matrix is set as zero. The correlation coefficient and each element of the adjacency matrix constructed by the first method are formulated as follows:

(12) $$\rho (X_{i},\;X_{j})=\frac{\mathrm{cov}(X_{i},\;X_{j})}{\sigma X_{i}\sigma X_{j}}$$

(13) $$A[i,\;j]=\left\{\begin{array}{c} 1,\;\rho (X_{i},\;X_{j})\geq \zeta \\ 0,\;\rho (X_{i},\;X_{j})<\zeta \end{array}\right.$$

where $\rho (X_{i},\;X_{j})$ is the Pearson correlation coefficient between the feature $X_{i}$ of the node *i* and the feature $X_{j}$ of the node *j*, $\mathrm{cov}(X_{i},\;X_{j})$ is the covariance of $X_{i}$ and $X_{j}$, $\sigma_{X_{i}}$ and $\sigma_{X_{j}}$ are the standard deviations of $X_{i}$ and $X_{j}$ respectively, A[i, j] is the *j*^th^ element of the *i*^th^ row in the adjacency matrix of the spatial graph, and ζ is the predefined threshold, which is set as 0.9 in this paper.

The second method for constructing the adjacency matrix is shown in Figure 4b. Specifically, in order to precisely quantify the correlation between the two nodes in the spatial graph, the corresponding element of the adjacency matrix is set as the correlation coefficient between the features of the two nodes. Thus, each element of the adjacency matrix constructed by the second method is formulated as follows:

(14) $$A[i,\;j]=\rho (X_{i},\;X_{j})$$

#### 3.3.2. Implementation Process

**Figure 5:**
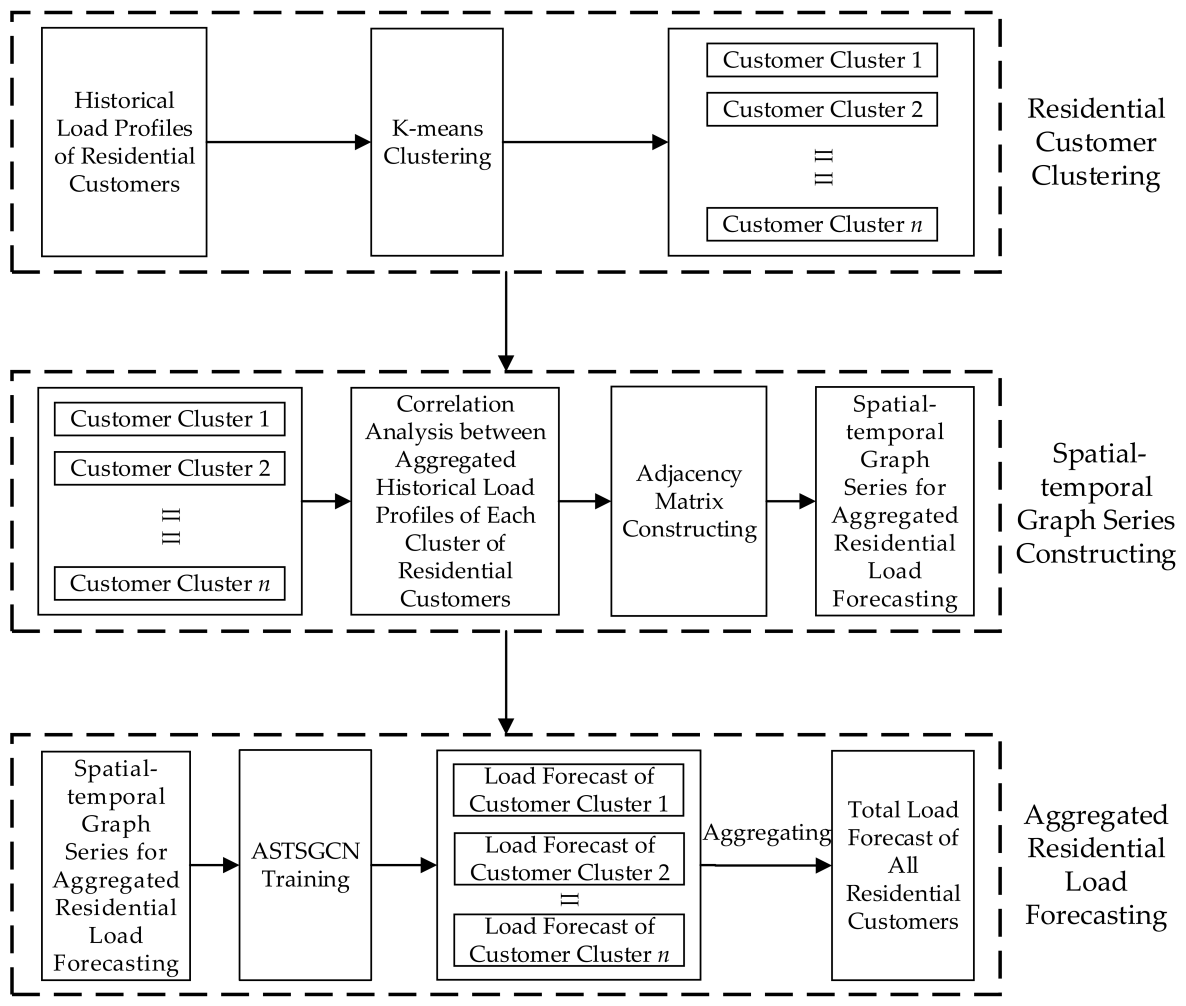
Flow chart of short-term aggregated residential load forecasting based on graph neural networks and K-means clustering.

Figure 5 shows the overall implementation process of short-term aggregated residential load forecasting based on graph neural networks and K-means clustering. Firstly, based on the similarity of electricity consumption behaviors of residential customers, K-means clustering is applied to divide the historical load profiles of all residential customers into groups. Secondly, according to the result of K-means clustering, the spatial–temporal graph series for short-term aggregated residential load forecasting is constructed. Then, an ASTSGCN is trained on the basis of the constructed spatial–temporal graph series in order to capture the electricity consumption pattern of each group of residential customers and forecast the load of each group of residential customers at the next time step. Finally, the load forecast of each group of residential customers is aggregated to obtain the total load forecast of all residential customers at the next time step.

## 4. Results and Discussion

### 4.1. Dataset Description

The dataset used in this paper is from the Smart Metering Electricity Customer Behaviour Trials initiated by Commission for Energy Regulation in Ireland [32]. The trials lasted from July 2009 to December 2010 with over 5000 Irish residential customers and small and medium enterprises participating. The sampling frequency of the dataset is once every half an hour. In this paper, 3639 residential customers with a complete record are selected as the experiment dataset. The whole dataset is chronologically divided into a training dataset, a validation dataset, and a test dataset with a ratio of 7:2:1. The training dataset and the validation dataset are used for training, while the test dataset is used for evaluation. Furthermore, the following normalization method is used for the training dataset, the validation dataset, and the test dataset respectively during data preprocessing:

(15) $$\hat{x}=\frac{x-\mathrm{mean}(X_{dataset})}{\mathrm{std}(X_{dataset})}$$

where $\hat{x}$ is the normalized value of the original load x of the residential customer, $\mathrm{mean}(X_{dataset})$ and $\mathrm{std}(X_{dataset})$ are the mean and the standard deviation of the dataset $X_{dataset}$ respectively.

### 4.2. Performance Evaluation Metrics

This paper utilizes mean absolute percentage error (MAPE), mean absolute error (MAE), and root mean squared error (RMSE) as evaluation metrics to assess the forecasting performance of different methods. These evaluation metrics are formulated as follows:

(16) $$\mathrm{MAPE}=\frac{1}{N}\sum_{i=1}^{N}|\frac{\hat{y_{i}}-y_{i}}{y_{i}}|\times 100\%$$

(17) $$\mathrm{MAE}=\frac{1}{N}\sum_{i=1}^{N}\left|\hat{y_{i}}-y_{i}\right|$$

(18) $$\mathrm{RMSE}=\sqrt{\frac{1}{N}\sum_{i=1}^{N}{\left(\hat{y_{i}}-y_{i}\right)}^{2}}$$

where $y_{i}$ is the real value of the load, $\hat{y_{i}}$ is the corresponding forecasted value of the load, and *N* is the number of samples of the test dataset.

### 4.3. Experiment Setup

This paper uses PyTorch 2.5.0 for model programming and conducts case studies on a standard desktop with an Intel Core i9-7920X CPU running at 2.90 GHz and 16 GB of RAM. As this paper is not focused on improving the forecasting performance via the optimal network structure, hyperparameter fine-tuning is not conducted on the ASTSGCN structure here. In addition, it is noted that each time step in the experiment is half an hour, and the input of the forecasting task of this paper is based on the load profiles of all residential customers at the previous time steps and its corresponding output is the forecasted aggregated residential load at the next time step. All the experiment parameters are presented in Table 1.

### 4.4. Experimental Results and Analysis

#### 4.4.1. Effect of Different Numbers of Clusters on Forecasting Performance

In order to explore the effect of different numbers of clusters on the forecasting performance of the proposed method, this paper set the number of clusters *K* as an integral value from 3 to 17 respectively to perform aggregated residential load forecasting. Table 2 describes the forecasting performance of the proposed method for different numbers of clusters and different adjacency matrices. It can be easily seen that as the number of clusters *K* changes from 3 to 17, the forecasting error of the proposed method firstly decreases and then increases as a whole, although there is a certain degree of fluctuation during the change in the number of clusters *K*. Moreover, it is noted that when the number of clusters *K* is 12, the proposed method obtains the minimum MAE, MAPE, and RMSE values. That is to say, the proposed method achieves the optimal forecasting performance when the number of clusters *K* is 12.

Furthermore, it is obvious in Table 2 that regardless of the number of clusters *K*, the second method for constructing the adjacency matrix can generally improve the forecasting performance of the proposed method, compared to the first method for constructing the adjacency matrix. To be specific, the main reason for that can be explained as follows. The second method can not only reflect whether there is a correlation relationship between different nodes through the Pearson correlation coefficient, but also precisely quantify the correlation relationship, therefore helping the proposed method to accurately capture the potential spatial–temporal features of the spatial–temporal graph series. However, the first method can only roughly reflect whether there is a correlation relationship between different nodes based on ones or zeros. For example, when the optimal number of clusters *K* is 12, based on the second method, the proposed method can obtain an MAE reduction of 1.5%, an MAPE reduction of 4.8%, and an RMSE reduction of 1.7%, compared to the first method.

#### 4.4.2. Performance Comparison of Short-Term Aggregated Residential Load Forecasting

In this paper, in order to verify the superiority of the proposed method (namely ASTSGCN), long short-term memory (LSTM), random forest (RF), support vector regression (SVR), and decision tree (DT) were selected as benchmark methods to compare with the proposed method. It is noted that the input of all benchmark methods is the historical aggregated load profile of all residential customers, while the output is the total load forecast of all residential customers at the next time step.

Table 3 presents the performance comparison of different forecasting methods. It is noted that the forecasting performance metrics of the proposed method refer to those obtained by the proposed method on the basis of the second method for constructing the adjacency matrix when the optimal number of clusters *K* is 12. As can be seen from Table 3, the proposed method obtains the best forecasting performance in terms of MAE, MAPE, and RMSE. In other words, compared to all benchmark methods, the proposed method obtains different reductions in the three forecasting performance metrics. For instance, compared to the LSTM method, which has the optimal forecasting performance among all benchmark methods, the proposed method can still obtain an MAE reduction of 23.3%, an MAPE reduction of 28.6%, and an RMSE reduction of 20.9%.

**Figure 6:**
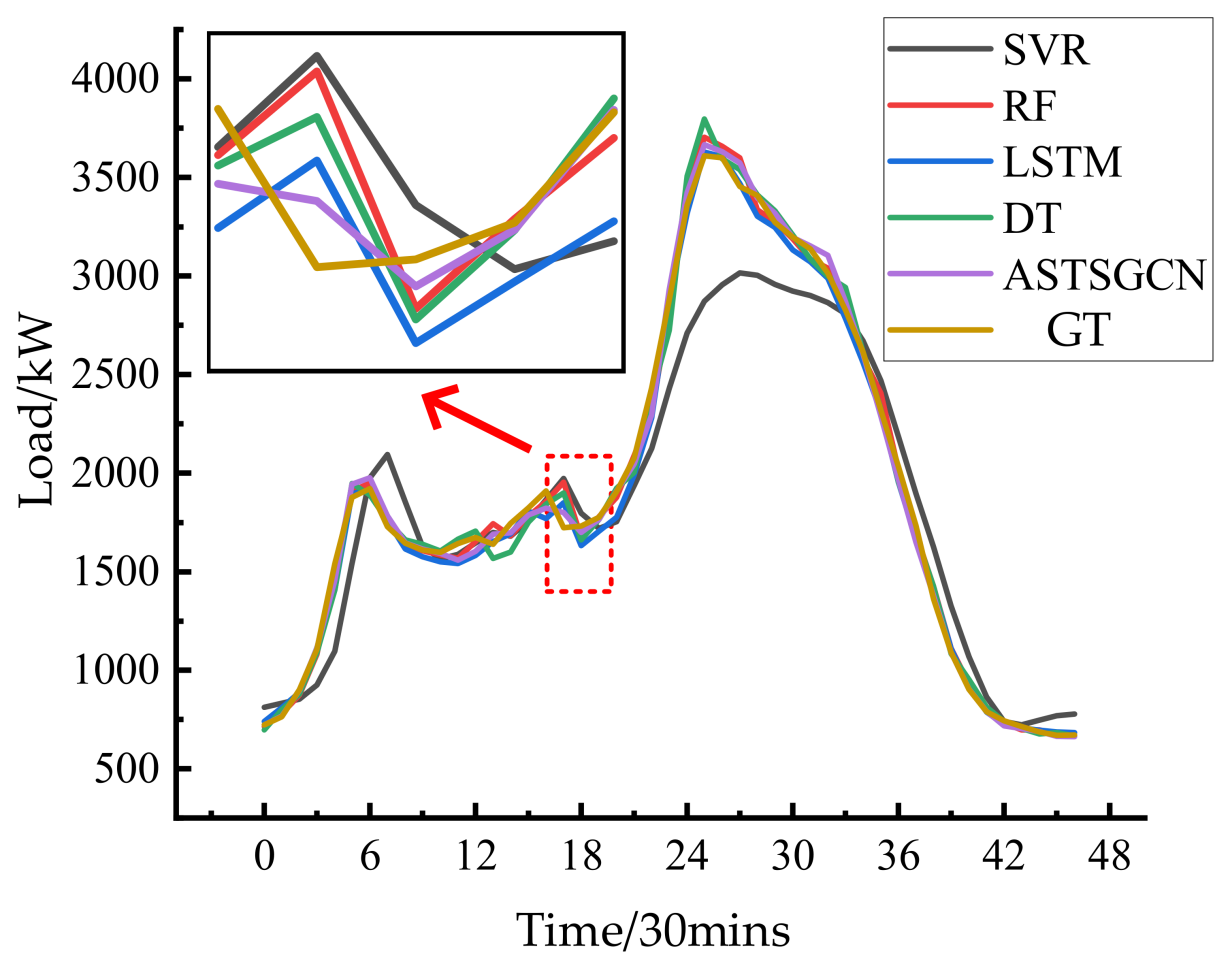
Aggregated load profiles of all residential customers by different forecasting methods on a certain day.

In addition, Figure 6 depicts the aggregated load profiles of all residential customers by different forecasting methods on a certain day. It is obvious in Figure 6 that compared to those of all benchmark methods, the forecasted aggregated load profile of the proposed method is much closer to the real aggregated load profile (namely GT). As a result, it is proved that the proposed method has the superior forecasting performance to all benchmark methods.

**Figure 7:**
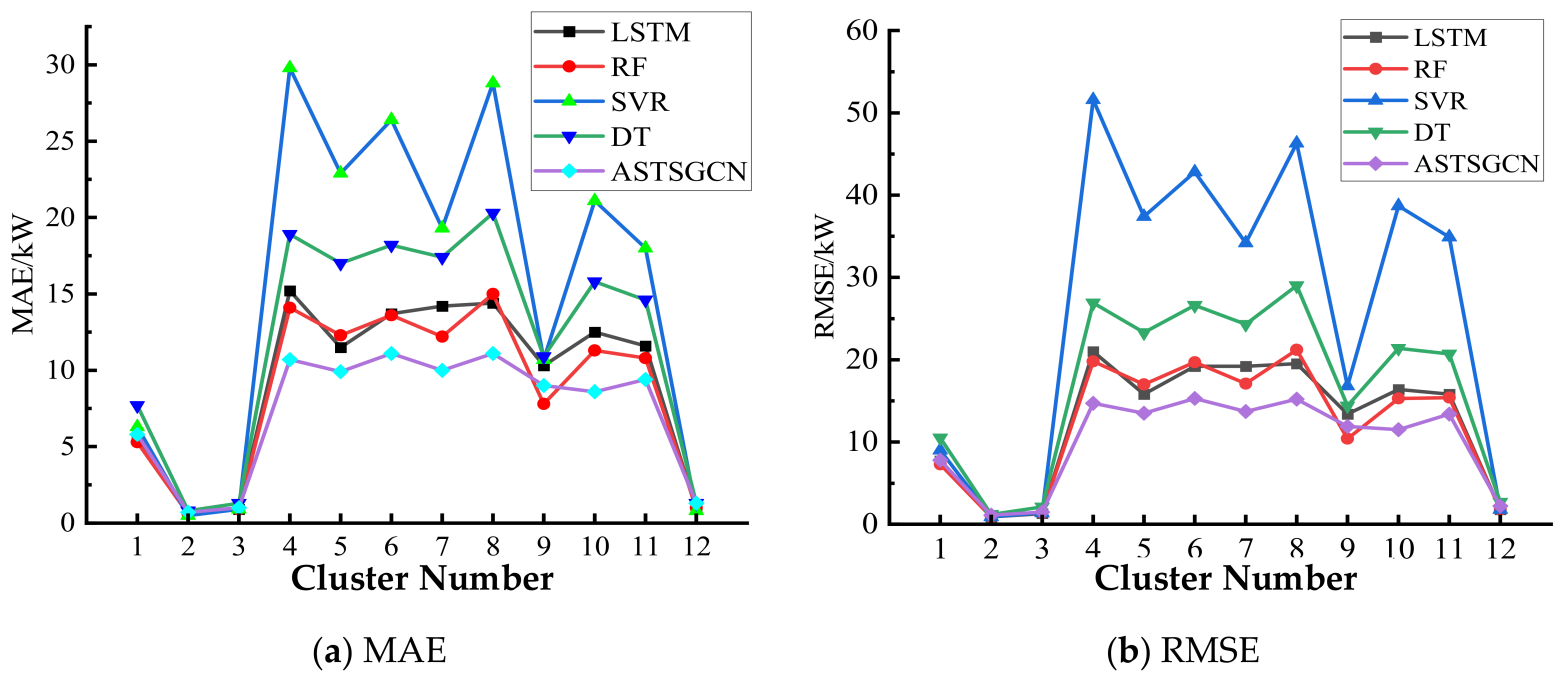
Forecasting performance comparison on the aggregated load of each cluster of residential customers (*K* = 12).

In order to further illustrate the performance improvement of the proposed method in terms of aggregated residential load forecasting, Figure 7 depicts the forecasting performance comparison of different methods on the aggregated load of each cluster of residential customers (*K* = 12). It can be clearly seen from Figure 7 that compared to all benchmark methods, the proposed method generally obtains different reductions in both MAE and RMSE regardless of each cluster, thereby decreasing the overall forecasting error. Hence, the proposed method has significant superiority over all benchmark methods in terms of aggregated residential load forecasting.

**Figure 8:**
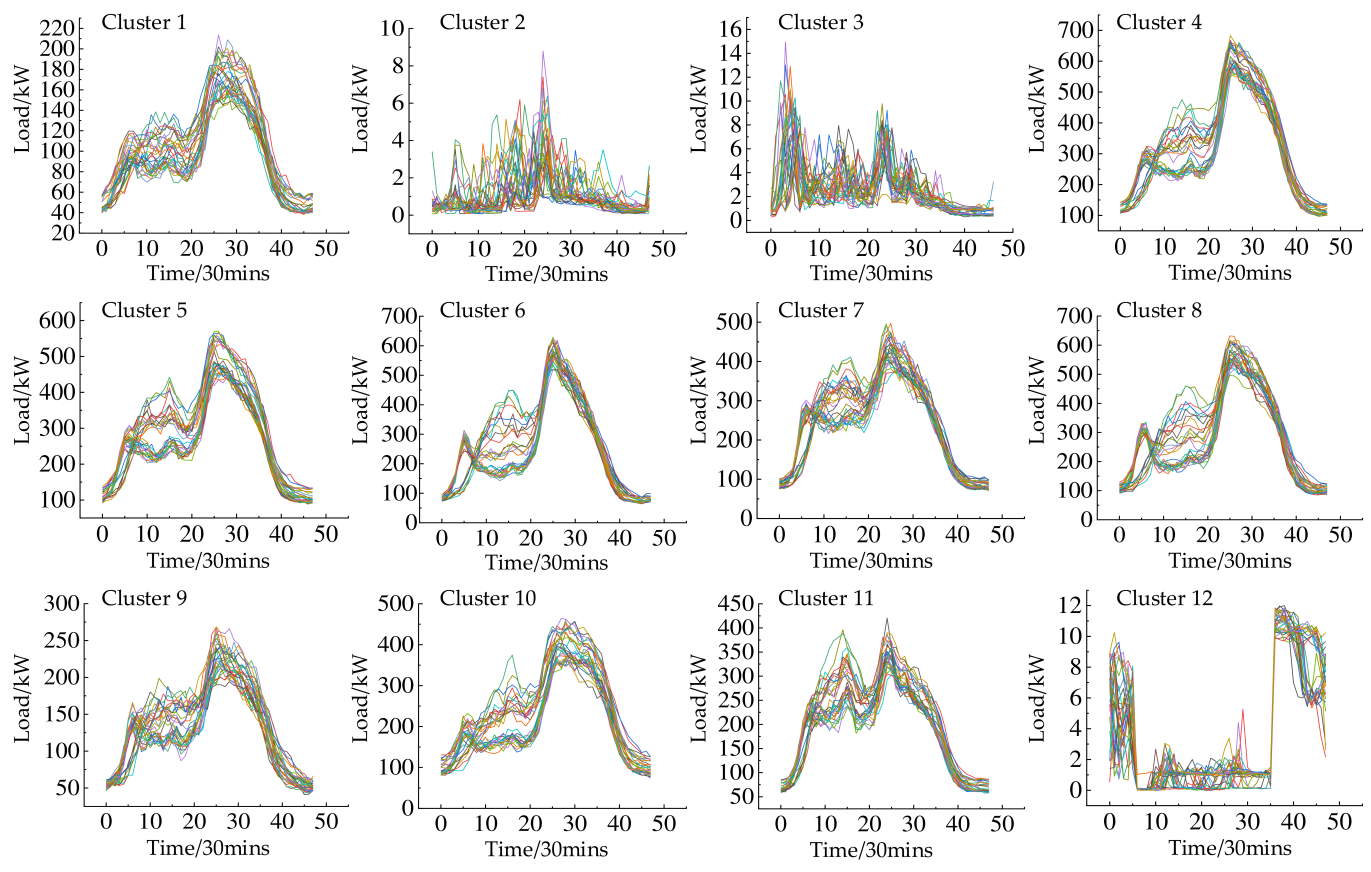
Daily aggregated load profiles of each cluster of residential customers in a certain month (*K* = 12).

Furthermore, in order to analyze the correlation between the electricity consumption pattern of each cluster of residential customers and the forecasting performance, Figure 8 presents the daily aggregated load profiles of each cluster of residential customers in a certain month (*K* = 12). As can be seen from Figure 8, the aggregated load of the residential customers of the first, fourth, fifth, sixth, seventh, eighth, ninth, 10th, and 11th clusters is much greater, and there is stronger regularity in the daily aggregated load profiles. Hence, the proposed method achieves a higher forecasting accuracy for those clusters. By comparison, the aggregated load of the residential customers of the second, third, and 12th clusters is relatively smaller, and there is higher volatility and uncertainty in the daily aggregated load profiles, therefore having a slightly negative effect on the forecasting accuracy of the proposed method. The main reason for this is that when the aggregated load of each cluster of residential customers has stronger regularity, the proposed method can improve the forecasting accuracy for each cluster effectively by capturing the spatial correlation between electricity consumption behaviors of different clusters of residential customers. Otherwise, it will affect the forecasting accuracy of the proposed method negatively. In addition, it is obvious in Figure 7 that for the first and ninth clusters, the forecasting accuracy of the proposed method is slightly lower than that of the RF method. That is primarily because there is a certain degree of fluctuation in the aggregated load of the residential customers of the first and ninth clusters, especially during the time period from the fifth time step to the thirty-fifth time step.

#### 4.4.3. Characteristic Analysis of Adaptive Adjacency Matrices

**Figure 9:**
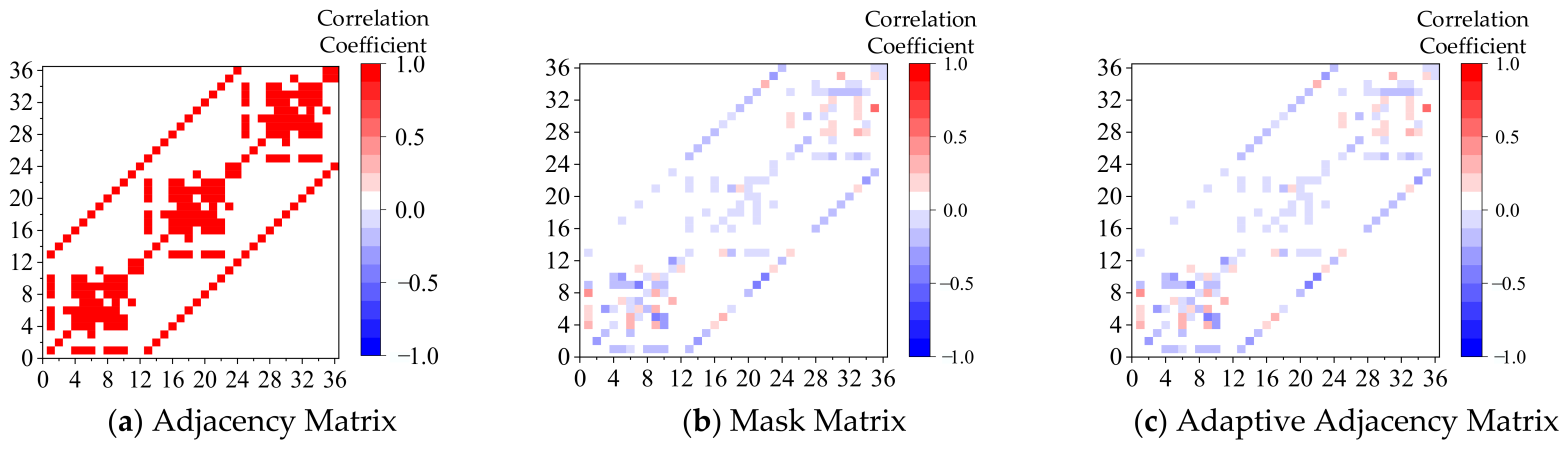
Adaptive adjacency matrix of the localized spatial–temporal graph obtained based on the first method (*K* = 12).

**Figure 10:**
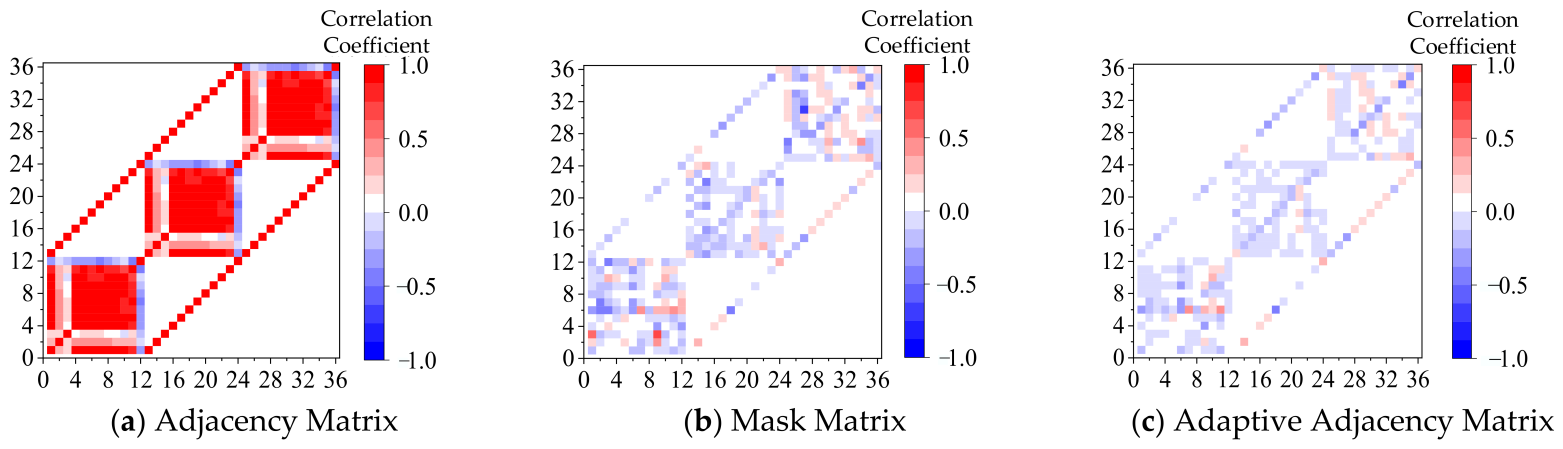
Adaptive adjacency matrix of the localized spatial–temporal graph obtained based on the second method (*K* = 12).

In order to further analyze the characteristics of the adaptive adjacency matrix of the localized spatial–temporal graph, the adjacency matrix of the spatial graph was constructed by the first and second methods respectively, and then the corresponding mask matrix was optimized based on training, finally obtaining the adaptive adjacency matrix of the localized spatial–temporal graph. Figure 9 and Figure 10 present the adaptive adjacency matrix of the localized spatial–temporal graph obtained based on the first and second methods respectively (*K* = 12). As can be seen clearly in Figure 9 and Figure 10, the spatial correlation among the first, fourth, fifth, sixth, seventh, eighth, ninth, 10th, and 11th clusters of residential customers is generally stronger, while the spatial correlation among the second, third, and 12th clusters of residential customers is comparatively weaker. In addition, although the adjacency matrix of the spatial graph constructed by the first method is quite different from that constructed by the second method, the adaptive adjacency matrices of the localized spatial–temporal graph finally obtained based on these two methods are almost the same, therefore effectively ensuring the accuracy of aggregated residential load forecasting. That is mainly because the proposed method can adaptively learn to obtain the corresponding mask matrix. Thus, it can be inferred that the proposed method is capable of adaptively learning to capture the potential spatial correlation between different nodes. Furthermore, it is obvious in Table 2 that compared to the first method, the second method utilizes the actual Pearson correlation coefficient to precisely characterize the potential spatial correlation between different nodes, thereby improving the forecasting accuracy. As a result, it can be inferred that the reasonably constructed adjacency matrix of the spatial graph can contribute to the improvement of the forecasting accuracy of the proposed method to some extent.

## 5. Conclusions

This paper proposed a short-term aggregated residential load forecasting method, which integrates graph neural networks with K-means clustering to effectively address the issue of spatial–temporal correlation in aggregated residential load forecasting. The proposed method combines K-means clustering with the Pearson correlation coefficient to construct the spatial–temporal graph series for aggregated residential load forecasting, and utilizes ASTSGCNs to extract both the temporal correlation of residential load and the spatial correlation between electricity consumption behaviors of adjacent residential customers. The effect of the number of clusters on the proposed method is explored, and the comparison results have shown that the optimal number of clusters can be found out to achieve the best forecasting performance. Moreover, in comparison with various benchmark methods, the proposed method has substantially improved the accuracy of aggregated residential load forecasting. The characteristic analysis of the adaptive adjacency matrix of the localized spatial–temporal graph is further conducted, and the experimental results have indicated that it can accurately characterize the potential spatial correlation between different nodes, therefore contributing to the improvement of the forecasting accuracy of the proposed method.

A future study will focus on different clustering techniques to combine with graph neural networks and explore their effects on aggregated residential load forecasting. A variety of hyperparameter fine-tuning techniques will also be further investigated to achieve the optimal forecasting performance. In addition, various internal and external factors, such as electric vehicles, distributed generation, weather, and electricity price, will be integrated as the explicit input features of aggregated residential load forecasting in order to further improve the forecasting performance.

## Figures and Tables

**Table 1:** Experiment parameters of short-term aggregated residential load forecasting based on graph neural networks and K-means clustering.

Parameter	Value
Time length of the historical spatial–temporal graph series	12
Neuron number of the input transform layer	64
Activation function of the input transform layer	ReLU
Number of ASTSGCLs	2
Number of ASTSGCMs	16
Number of graph convolutional layers	4
Time length of the sliding window	3
Activation function of graph convolutional layers	ReLU
Number of the output transform layers	2
Neuron number of the output transform layers	128
Activation function of the output transform layers	ReLU
Time length of the future spatial–temporal graph series	1
Learning rate	0.003
Optimization function	Adam
Loss function	Huber
Learning rate decay	0.3
Batch size	32
Training epoch	100

**Table 2:** Forecasting performance of the proposed method for different numbers of clusters and different adjacency matrices ^1^.

Number of Clusters	MAE (kW)		MAPE (%)		RMSE (kW)	
	0–1	ρ	0–1	ρ	0–1	ρ
K = 3	49.7	49	2.3	2.2	72.5	69.3
K = 4	49.8	49.9	2.3	2.3	71.9	71.1
K = 5	52.5	51	2.4	2.3	76.2	72.2
K = 6	58.4	51.6	2.6	2.4	74.4	72.4
K = 7	52.4	51.3	2.5	2.3	71.7	71.8
K = 8	58.4	49.1	2.8	2.3	79.0	68.5
K = 9	49.7	45.8	2.2	2.1	70.7	64.2
K = 10	47.9	47.1	2.2	2.2	66.4	65
K = 11	49.7	46.2	2.3	2.1	68.9	64.7
K = 12	45.5	44.8	2.1	2.0	64.1	63
K = 13	48.5	47.7	2.2	2.2	69.9	66.8
K = 14	47.6	47.5	2.2	2.2	65.9	65.9
K = 15	49.6	49.5	2.4	2.3	68.3	70.0
K = 16	48.4	45.1	2.3	2.1	66.6	63.4
K = 17	53.2	48.6	2.4	2.2	76.0	68.9

^1^ 0–1 denotes the first method for constructing the adjacency matrix, and ρ denotes the second method for constructing the adjacency matrix.

**Table 3:** Performance comparison of different forecasting methods.

Forecasting Methods	MAE (kW)	MAPE (%)	RMSE (kW)
LSTM	58.4	2.8	79.6
RF	70.1	3.1	102.6
SVR	158.9	6.2	280.5
DT	69.7	3.1	99.6
ASTSGCN	44.8	2.0	63.0

## Data Availability

Restrictions apply to the availability of these data. Data were obtained from Commission for Energy Regulation in Ireland and are available at https://issda.ucd.ie/dataset.xhtml?persistentId=doi:10.7929/ISSDA/BX59EU (accessed on 21 March 2018) with the permission of Commission for Energy Regulation in Ireland.

## Abbreviations

The following abbreviations are used in this manuscript:

ASTSGCN	Adaptive spatial–temporal synchronous graph convolutional network
ASTSGCL	Adaptive spatial–temporal synchronous graph convolutional layer
ASTSGCM	Adaptive spatial–temporal synchronous graph convolutional module
MAPE	Mean absolute percentage error
MAE	Mean absolute error
RMSE	Root mean squared error
LSTM	Long short-term memory
RF	Random forest
SVR	Support vector regression
DT	Decision tree
GT	Ground truth
